# I - Avaliação da Função Autonômica Cardiovascular em Indivíduos Saudáveis

**DOI:** 10.36660/abc.20250111

**Published:** 2026-01-09

**Authors:** Benedito Carlos Maciel, Lourenço Gallo-Jr, André Schmidt, José Antonio Marin-Neto

**Affiliations:** 1 Hospital das Clínicas Faculdade de Medicina de Ribeirão Preto USP São Paulo SP Brasil Laboratório de Hemodinâmica e Cardiologia Intervencionista, Hospital das Clínicas, Faculdade de Medicina de Ribeirão Preto, USP, São Paulo, SP – Brasil

**Keywords:** Doenças do Sistema Nervoso Autônomo, Frequência Cardíaca, Sistema Nervoso Simpático, Sistema Nervoso Parassimpático

## Abstract

Esta primeira parte da revisão apresenta quase 60 anos de experiência de nosso laboratório na padronização de testes autonômicos cardiovasculares em indivíduos saudáveis. A ampla gama de testes inclui bloqueio farmacológico dos sistemas simpático e parassimpático, avaliação da sensibilidade barorreflexa por meio de episódios transitórios de hipertensão e hipotensão, manobra de Valsalva, teste de inclinação ortostática, exercícios isométricos e dinâmicos, imersão facial em água fria, arritmia sinusal respiratória, análise da variabilidade da frequência cardíaca no domínio da frequência e exames de imagem com ^123^I-metaiodobenzilguanidina. A função autonômica foi avaliada com base nas respostas aos testes em condições basais em indivíduos saudáveis. A análise também aborda adaptações fisiológicas ao treinamento de resistência e ao envelhecimento, fornecendo uma estrutura de referência para a identificação de disfunções autonômicas envolvendo componentes parassimpáticos e adrenérgicos em pacientes com diversas condições fisiopatológicas, conforme discutido na segunda parte desta revisão.

## Graphical abstract

**Figura Central:**
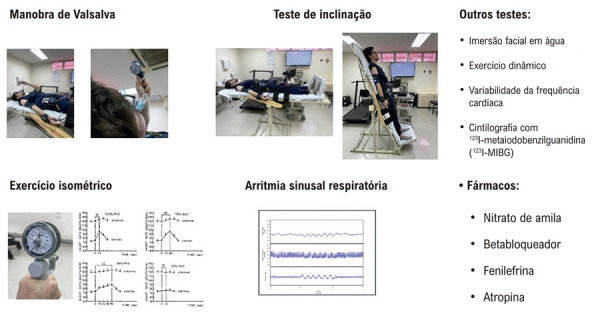
Avaliação da Função Autonômica Cardiovascular em Indivíduos Saudáveis (I)

## Introdução

Em indivíduos saudáveis, uma complexa rede neural autonômica, tanto anatômica quanto funcional, regula as funções cronotrópica, lusitrópica, dromotrópica e inotrópica do coração.^1,2^ Esta primeira parte da revisão reúne os achados de estudos realizados em indivíduos saudáveis. O conhecimento derivado desses estudos constitui a base para compreender como a disfunção do sistema nervoso autonômico cardiovascular contribui para a fisiopatologia de diversas condições clínicas. Os achados relacionados a tais disfunções em condições anormais são discutidos na segunda parte desta publicação.

Nesta revisão, baseamo-nos em mais de 55 anos de experiência de nosso laboratório com o uso de testes padronizados para avaliar a fisiologia autonômica cardíaca em indivíduos saudáveis e a disfunção autonômica em humanos com diversas condições fisiopatológicas. Embora a função autonômica cardiovascular possa ser avaliada por meio de registros diretos de nervos ou técnicas baseadas em imagem — como a captação ventricular de ^123^I-metaiodobenzilguanidina (^123^I-MIGB) —, nosso trabalho concentrou-se principalmente nas respostas da frequência cardíaca (FC) a diversos estímulos mediados pelas influências autonômicas sobre o nó sinusal.

A ampla gama de testes examinou diferentes aspectos do controle autonômico cardíaco em condições de repouso, durante bloqueio farmacológico dos ramos parassimpático e/ou simpático do sistema nervoso autonômico e em resposta a manobras fisiológicas padronizadas, conforme descrito a seguir.

Nos diferentes estudos, os grupos de indivíduos saudáveis variaram de 8 a 23 participantes, com idade média entre 27 e 31 anos (faixa etária: 19 a 43 anos). Todos os participantes eram do sexo masculino e foram considerados saudáveis após uma avaliação clínica abrangente. Nenhum deles fazia uso de medicações no momento dos testes.

### Testes funcionais para avaliação da função autonômica cardiovascular

Para avaliar a função autonômica cardiovascular em indivíduos saudáveis, padronizamos o uso de testes em condições basais de repouso e após bloqueio farmacológico dos ramos eferentes parassimpático e simpático do sistema nervoso autonômico (Figura Central). Esses testes também incluíram a avaliação dos reflexos da FC em resposta a alterações fisiológicas e farmacológicas da pressão arterial sistólica (PAS).

Resumidamente, após o registro basal da FC e da PAS com o indivíduo em posição supina (geralmente após 20 minutos de repouso), os mesmos parâmetros foram medidos após administração intravenosa de: a) sulfato de atropina (0,04 mg/kg, infundido lentamente a 0,25 mg/min); ou b) cloridrato de propranolol (0,2 mg/kg, infundido lentamente a 1 mg/min).

Na maioria dos estudos, a FC e a PAS também foram registradas após a administração combinada de atropina e propranolol por via intravenosa, permitindo a determinação da FC intrínseca do nó sinusal. As manobras fisiológicas descritas nas seções seguintes desta revisão foram realizadas antes e depois de cada um desses três tipos de bloqueio autonômico farmacológico.

### Avaliação do controle barorreflexo da FC: fenilefrina, nitrito de amila e manobra de Valsalva^3^

Todos os testes foram realizados com os participantes em posição supina. Sob anestesia local, foi inserido um cateter venoso na veia antecubital e uma agulha Cournand 20G foi posicionada na artéria braquial. Um bolus intravenoso de 25 a 400 µg de cloridrato de fenilefrina foi administrado, seguido por lavagem com 10 ml de solução salina, com o objetivo de induzir um aumento de 20 a 30 mmHg na PAS. Para o teste com nitrito de amila, o conteúdo de duas ampolas (0,2 ml cada, 96% de nitrito de amila) foi inalado ao longo de dois ciclos respiratórios, com a meta de reduzir a PAS entre 10 e 30 mmHg abaixo do valor basal.

A sensibilidade barorreflexa foi avaliada por meio da análise das alterações reflexas agudas da FC, desencadeadas por variações transitórias da PAS, conforme descrito a seguir.

A manobra de Valsalva foi realizada por meio de uma expiração forçada contra resistência, garantindo que a glote permanecesse aberta durante o esforço. A pressão expiratória foi mantida em 40 mmHg por 20 segundos. Durante os testes com fenilefrina, nitrito de amila e a manobra de Valsalva, ecocardiografia (ECG), PAS e pressão oral foram registrados continuamente em velocidade de papel de 25 mm/s.

Os dados obtidos no teste com fenilefrina foram analisados selecionando-se, para cada indivíduo, apenas as injeções que geraram linhas de regressão entre cada valor de PAS e o intervalo RR subsequente no ECG, desde que os coeficientes de correlação fossem estatisticamente significativos (p < 0,05). As inclinações de 3 a 4 linhas de regressão foram então calculadas para obter a sensibilidade barorreflexa média de cada indivíduo, segundo o método descrito por Smith, Sleight et Pickering.^4^ A sensibilidade barorreflexa durante o teste com nitrito de amila foi quantificada traçando os valores sequenciais decrescentes da PAS contra os intervalos RR correspondentes do ciclo cardíaco seguinte. A média de três testes foi calculada para cada indivíduo.^4-6^

As alterações da PAS durante a manobra de Valsalva foram registradas continuamente ao longo de todas as fases do teste. A manobra provoca uma redução súbita da pré-carga cardíaca, devido à diminuição do retorno venoso, resultando em queda da PAS e ativando mecanismos compensatórios, como vasoconstrição arterial e taquicardia durante o esforço expiratório. Em indivíduos saudáveis, observa-se tipicamente uma recuperação parcial da PAS ainda durante a fase de esforço.^7^ A resposta cronotrópica foi expressa pela razão de Valsalva, calculada pela razão entre o maior intervalo RR observado na fase IV (pós-esforço, quando ocorre o aumento de pressão) e o menor intervalo RR verificado durante a fase II (fase hipotensiva do esforço expiratório). A média de três testes foi registrada para cada indivíduo.^6^

Todos os testes foram realizados antes e depois da administração intravenosa de sulfato de atropina, conforme descrito anteriormente. A magnitude do aumento da FC após a administração de atropina reflete o nível de inibição vagal tônica sobre o nó sinusal.

Uma visão adicional sobre a influência parassimpática no coração é fornecida pelos valores de sensibilidade barorreflexa medidos durante episódios transitórios de hipertensão e hipotensão induzidos farmacologicamente — por fenilefrina e nitrito de amila, respectivamente. Durante a manobra de Valsalva, a função autonômica parassimpática é avaliada principalmente pela bradicardia que ocorre imediatamente após o fim do esforço expiratório (fase IV), o que leva a uma redução transitória da FC abaixo do valor basal. Importante notar que o aumento precoce da FC observado durante a fase II não decorre da ativação simpática, mas sim da retirada parassimpática.^7^ A resposta adrenérgica vasomotora é refletida na recuperação da pressão arterial durante as fases III e IV da manobra de Valsalva. Paralelamente à retirada parassimpática, o aumento da FC durante a fase de esforço (fase II) também representa a intensidade da ativação simpática atuando sobre o nó sinusal.^3^

### Teste de inclinação para avaliar o controle da FC pelos sistemas parassimpático e adrenérgico

Para avaliar os efeitos hemodinâmicos do estresse ortostático agudo sobre a regulação da FC e da PAS, indivíduos saudáveis foram inclinados de forma rápida e passiva para uma posição ortostática de 70°, após permanecerem 20 minutos em posição supina. Utilizou-se uma mesa de inclinação especializada para eliminar qualquer esforço muscular, independentemente da posição corporal. A FC instantânea foi monitorada por meio de um medidor linear de frequência ativado por uma derivação padrão do ECG, com ambos os sinais sendo exibidos e registrados em tempo real por um sistema de aquisição online. A PAS foi registrada continuamente após a inserção percutânea de uma agulha de Cournand na artéria braquial ou femoral, conectada a um transdutor de pressão com medidor de deformação.^8^ Os parâmetros hemodinâmicos foram registrados enquanto os indivíduos permaneciam passivamente inclinados a 70° por 5 minutos.

O procedimento foi então repetido após administração intravenosa de sulfato de atropina (0,04 mg/kg, infundido lentamente a 0,25 mg/min). Em uma sessão subsequente, realizada em outro dia, após o término dos efeitos da atropina, a mudança postural para a posição ortostática foi repetida sob o efeito do cloridrato de propranolol (0,2 mg/kg, infundido lentamente a 1 mg/min). Dentro de 60 minutos após a administração do propranoloWl, a atropina foi novamente administrada conforme descrito anteriormente. Um terceiro teste de inclinação foi então realizado para avaliar as respostas hemodinâmicas ao estresse postural sob duplo bloqueio autonômico — isto é, com a FC intrínseca do nó sinusal em ação, sem modulação autonômica.

O estresse ortostático provoca acúmulo agudo de sangue no sistema venoso dos membros inferiores, tanto quando os indivíduos assumem ativamente a posição ereta quanto quando são passivamente inclinados a 70°. Essa manobra leva à redução do retorno venoso ao coração e a uma queda rápida de 15 a 25% no volume sistólico e no débito cardíaco. Em indivíduos saudáveis, mecanismos fisiológicos compensatórios incluem taquicardia reflexa e vasoconstrição arteriolar sistêmica. Após uma breve e leve queda da PAS, estabelece-se tipicamente um estado hemodinâmico estável, com a PAS média mantida em níveis semelhantes aos observados em posição supina. A redução do volume sistólico é, portanto, compensada por um aumento da FC e pela elevação da resistência vascular sistêmica.^7,8^

Em indivíduos saudáveis, observa-se um aumento significativo da FC — de aproximadamente 15 a 25 batimentos por minuto — nos primeiros 10 segundos após a inclinação passiva para a posição ereta. Essa resposta inicial é mediada pela retirada parassimpática, sendo abolida após a administração de atropina. O bloqueio beta-adrenérgico não afeta significativamente esse aumento inicial da FC (nos primeiros 10 segundos); no entanto, reduz de forma marcante a elevação da FC observada após 1 e 5 minutos de inclinação em indivíduos saudáveis. Esses achados sugerem uma resposta taquicárdica bifásica ao estresse ortostático: a fase inicial é predominantemente mediada pela retirada parassimpática, enquanto a ativação simpática passa a ser o principal mecanismo após o estabelecimento da estabilização hemodinâmica na posição ereta.

### Imersão facial em água como teste do controle parassimpático da FC

Estudos prévios demonstraram que a bradicardia induzida pelo mergulho ou pela imersão facial é mediada pela via eferente parassimpática, uma vez que pode ser abolida por vagotomia cirúrgica ou administração de atropina.^9,10^ Embora diversos receptores e vias aferentes possam contribuir para essa resposta, o impacto relativo de estímulos individuais — como a própria imersão, a temperatura da água, o volume pulmonar, a apneia e a pressão intratorácica — ainda não foi totalmente esclarecido.

Em nosso laboratório, investigamos a resposta da FC à imersão facial em água, tanto como manobra isolada quanto em combinação com apneia, em participantes saudáveis. O objetivo foi definir as condições sob as quais essa resposta poderia ser utilizada como um teste padronizado, simples, não invasivo e reprodutível da atividade parassimpática.

Foram avaliados os seguintes procedimentos:^11^ (a) apneia de 10 segundos em ar, em diferentes volumes pulmonares; (b) imersão facial em água por 2 minutos a diferentes temperaturas (5°C, 15°C e 25°C), com a respiração mantida por meio de um circuito aéreo fechado; e (c) combinação de imersão facial e apneia em diferentes volumes pulmonares. Um subgrupo de participantes saudáveis foi reavaliado após bloqueio farmacológico parassimpático com sulfato de atropina (0,04 mg/kg de peso corporal).

Nossos principais achados foram os seguintes: (a) a apneia em ar provocou respostas de FC dependentes do volume pulmonar; (b) a imersão facial em água induziu bradicardia transitória, com efeito máximo entre 20 e 30 segundos de imersão; (c) a temperatura da água não teve efeito apreciável sobre a magnitude da bradicardia; (d) a combinação de imersão e apneia produziu respostas heterogêneas da FC, sem qualquer potencialização da bradicardia em comparação com cada manobra isoladamente; e (e) a atropina não foi capaz de reduzir a magnitude da bradicardia induzida pela imersão facial em dois dos três indivíduos reavaliados após bloqueio parassimpático.

A variabilidade das respostas observadas neste estudo provavelmente se deve à estimulação simultânea de múltiplos receptores e vias aferentes durante as manobras. Como resultado, a resposta autonômica eferente depende da interação complexa e imprevisível entre esses mecanismos. Essa variabilidade representa um fator limitante para a padronização da imersão facial como método simples e reprodutível para avaliação da atividade parassimpática.^11^

Além disso, os resultados obtidos sob bloqueio farmacológico sugerem que a via eferente vagal não é o único fator responsável pela bradicardia induzida pela imersão facial sem apneia.^11^

### Regulação autonômica da FC durante exercício isométrico (EI) em indivíduos saudáveis

O padrão de resposta da FC ao EI sustentado tem sido amplamente estudado e encontra-se bem caracterizado em humanos, com base em pesquisas anteriores corroboradas por nossos próprios achados. A FC aumenta muito rapidamente no início da contração isométrica, geralmente nos primeiros 500 milissegundos de esforço. A magnitude desse aumento parece ser diretamente proporcional ao nível relativo de tensão muscular gerada — isto é, à porcentagem da contração voluntária máxima (CVM) que o indivíduo consegue sustentar por um período limitado com determinado grupo muscular (por exemplo, membros superiores ou inferiores). Quando a contração é mantida, a FC continua a subir progressivamente, especialmente em níveis mais elevados de tensão muscular.^12-14^

A contribuição relativa de cada ramo eferente do sistema nervoso autonômico para a taquicardia induzida pelo EI foi avaliada em indivíduos saudáveis por meio do método de preensão manual. O exercício foi realizado na CVM — definida como o maior nível de tensão que o indivíduo conseguia sustentar por 10 segundos — e em níveis submáximos de 75%, 50% e 25% da CVM, sustentados por 20, 40 e 10 segundos, respectivamente.^15^ Os indivíduos eram mantidos sentados, com a mão direita posicionada para segurar a alça de um dinamômetro isométrico. Os padrões respiratórios eram monitorados cuidadosamente para garantir respiração espontânea e regular por meio de um bocal conectado a uma válvula tipo Hans Rudolph, com oclusão nasal mantida por um clipe nasal. A CVM (considerada como 100%) foi estabelecida individualmente e utilizada para calibrar os demais níveis de intensidade testados.

**Figura 1:**
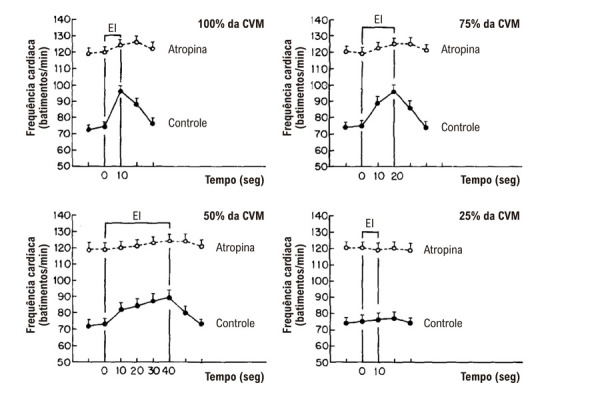
– Respostas da frequência cardíaca ao exercício isométrico em 100%, 75%, 50% e 25% da contração voluntária máxima em indivíduos saudáveis, durante períodos de esforço de 10, 20, 40 e 10 segundos, respectivamente, registrados em condições controle e após bloqueio farmacológico parassimpático com atropina (0,04 mg/kg de peso corporal; n = 12). Os valores são expressos como média ± EPM, em intervalos de 10 segundos. O período de exercício está indicado pelas barras verticais.15 EPM: erro padrão da média.

**Figura 2:**
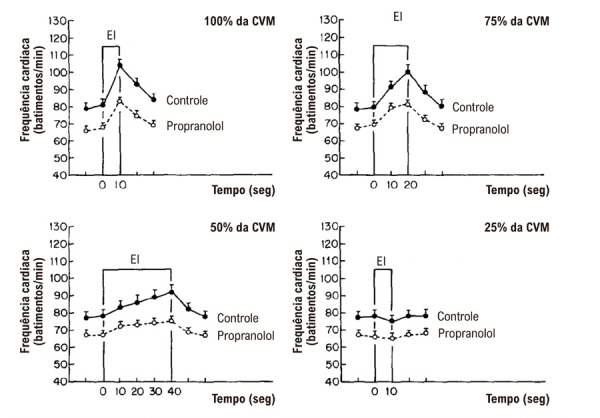
– Respostas da frequência cardíaca ao exercício isométrico em 100%, 75%, 50% e 25% da contração voluntária máxima em indivíduos saudáveis, durante períodos de esforço de 10, 20, 40 e 10 segundos, respectivamente, registrados em condições controle e após bloqueio farmacológico simpático com propranolol (0,2 mg/kg de peso corporal; n = 11). Os valores são apresentados como média ± EPM em intervalos de 10 segundos. O período de exercício está indicado pelas barras verticais.15 EPM: erro padrão da média.

O estudo foi conduzido tanto em condições basais quanto após bloqueio farmacológico com atropina ou propranolol.^15^ Em condições de controle, as respostas da FC ao EI foram dependentes da intensidade e da duração, apresentando aumento progressivo durante a contração sustentada. A taquicardia foi de início rápido e de magnitude substancial, especialmente em intensidades mais elevadas da preensão manual.^15^ O bloqueio parassimpático atenuou significativamente o aumento da FC observado nos primeiros 10 segundos de EI em todos os níveis de intensidade (Figura 1). Em contraste, o bloqueio simpático com propranolol reduziu de forma acentuada a resposta da FC após 10 segundos de esforço a 75% e 50% da CVM (Figura 2). Uma leve redução da taquicardia também pôde ser observada após apenas 10 segundos de EI máximo sob bloqueio simpático. Esses achados indicam que a regulação autonômica da FC durante o EI segue um padrão bifásico: a resposta inicial é mediada principalmente pela retirada rápida do tônus parassimpático, seguida por uma contribuição simpática mais proeminente, que se torna mais evidente após 10 segundos de contração sustentada — ou até um pouco antes, em intensidades máximas.^15^

### Regulação autonômica da FC durante exercício dinâmico (ED) em indivíduos saudáveis

Estudos prévios demonstraram que o início do ED é acompanhado por um aumento rápido da FC. A natureza abrupta dessa taquicardia sugere um mecanismo regulador de origem neurogênica, com a resposta eferente predominantemente mediada pela retirada vagal ao nível do nó sinusal. Após essa fase inicial, a FC continua a subir de forma mais gradual.^16-18^ Em nossas investigações com indivíduos do sexo masculino saudáveis, avaliamos as contribuições relativas dos ramos simpático e parassimpático do sistema nervoso autonômico na resposta de FC induzida pelo ED. O teste de exercício foi realizado antes e após bloqueio farmacológico com atropina ou propranolol. O ED foi executado em posição sentada, utilizando-se um cicloergômetro eletromagnético calibrado. Os indivíduos se exercitaram em cargas de trabalho de 25, 50 e 100 watts — cada uma sustentada por 4 minutos. Intervalos de repouso variáveis foram fornecidos entre os diferentes níveis de esforço para permitir o retorno da FC ao valor basal. A cadência de pedalada foi mantida constante em 60 rotações por minuto. O ECG e a FC instantânea foram registrados continuamente desde 30 segundos antes do início do exercício até 1 minuto após sua finalização.

**Figura 3:**
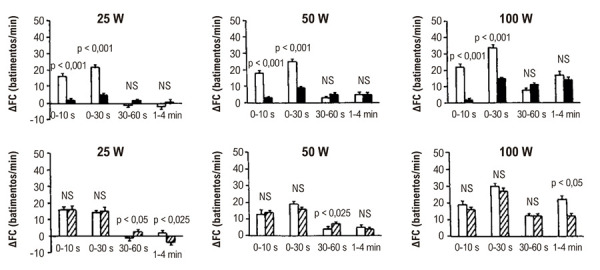
– Incrementos da frequência cardíaca (ΔFC) induzidos por exercício dinâmico em indivíduos saudáveis, com cargas de trabalho de 25, 50 e 100 watts, medidos nos seguintes intervalos de tempo: 0-10 s, 0-30 s, 30-60 s e 1-4 min. As barras representam os valores médios ± EPM registrados antes (barras vazadas) e após bloqueio farmacológico com atropina (0,04 mg/kg de peso corporal; n = 13, barras preenchidas) ou propranolol (0,2 mg/kg de peso corporal; n = 10, barras hachuradas). NS: não significativo.19 EPM: erro padrão da média.

O bloqueio parassimpático atenuou significativamente a resposta rápida da FC no início do exercício em todos os níveis de intensidade, enquanto o bloqueio simpático afetou principalmente o aumento mais lento da FC durante a fase de 1 a 4 minutos, especialmente na carga de trabalho mais elevada^19^ (Figura 3). Esses achados sustentam a hipótese de que a taquicardia induzida pelo ED é mediada por um mecanismo bifásico: uma fase inicial impulsionada por retirada vagal rápida, seguida por um aumento gradual da atividade simpática, que se torna mais pronunciado em intensidades mais altas de exercício.

Assim, o monitoramento contínuo da FC durante todo o período do ED, em diferentes intensidades, oferece um método simples e não invasivo para a avaliação funcional dos componentes parassimpático e simpático da regulação cardíaca autonômica.^19^

### Estudos combinando ED e EI em indivíduos saudáveis

Nossas investigações também incluíram indivíduos saudáveis realizando a combinação de EI e ED. Este estudo exemplifica a abordagem do nosso laboratório em integrar múltiplos testes autonômicos para melhor compreender as respostas fisiológicas humanas. O objetivo principal foi utilizar o EI como ferramenta para avaliar os mecanismos subjacentes ao aumento da FC durante o ED.

A intensidade do EI (preensão manual) foi padronizada para provocar aumento da FC principalmente por meio da retirada vagal. O EI foi realizado com um dinamômetro com medidor de deformação, de resposta linear, a 75% da CVM, sustentado por 10 segundos e repetido a cada 1 minuto. As respostas da FC ao EI foram avaliadas em condições basais, durante o ED e ao longo da fase de recuperação correspondente.

O ED foi realizado com os membros inferiores, em posição sentada, em um cicloergômetro eletromagnético por 4 minutos, com cargas de 55 e 105 watts, intercaladas com períodos de repouso. No protocolo combinado, o EI foi aplicado no início do ED, aos 1, 2 e 3 minutos durante o ED, e aos 0, 1, 2, 3 e 5 minutos da fase de recuperação.

Os seguintes resultados foram observados: (1) o EI realizado durante o ED produziu consistentemente um aumento menor da FC em comparação ao EI isolado. Esse efeito foi mais evidente com carga de 105 W do que com 55 W, sugerindo que a retirada vagal ocorreu logo no início do ED e foi sustentada ao longo do esforço de forma dependente da carga de trabalho; (2) durante o ED, o EI continuou a provocar aumentos adicionais da FC — mesmo na carga de 105 W, quando a FC já estava se elevando gradualmente apenas devido ao ED —, indicando que a taquicardia progressiva observada entre o primeiro e o quarto minuto do ED não era atribuível à retirada vagal; (3) o protocolo padronizado de EI permitiu a avaliação funcional da dinâmica da retirada vagal durante o ED sem a necessidade de bloqueio autonômico farmacológico.^20^

### Arritmia sinusal respiratória como teste do controle autonômico da FC

Como as variações da FC durante a arritmia sinusal respiratória são mediadas principalmente pela via eferente vagal,^21,22^ padronizamos esse método como uma técnica simples e quantificável para avaliar a atividade parassimpática.^23^Nesse teste, os indivíduos eram mantidos sentados e instruídos a respirar em um espirômetro de circuito fechado equipado com um potenciômetro linear. Esse sistema permitia o registro simultâneo do volume respiratório por meio de um registrador térmico e de um canal de um osciloscópio de memória.

O segundo canal do osciloscópio era conectado a um gerador de funções que produzia uma onda senoidal com amplitude e frequência predeterminadas. Os indivíduos eram instruídos a sincronizar seu padrão respiratório com o sinal senoidal gerado. O teste era realizado com frequência de 6 ciclos por minuto, e o volume corrente era ajustado para 1 litro. Essa padronização provocava de forma confiável uma arritmia sinusal respiratória acentuada em indivíduos saudáveis.

Durante todo o procedimento, o ECG, a FC instantânea e o volume corrente respiratório eram registrados continuamente. Para cada indivíduo, a variabilidade média da FC (VFC) foi calculada como a diferença entre a FC instantânea máxima e mínima ao longo de 9 ciclos respiratórios consecutivos.

### VFC como teste da função autonômica

Em indivíduos saudáveis, a VFC reflete principalmente a modulação autonômica eferente do nó sinusal. Durante muitos anos, a VFC foi expressa exclusivamente por medidas no domínio do tempo, como valores médios e desvios padrão, que quantificam a variabilidade com base nas flutuações dos intervalos RR. No entanto, a avaliação não invasiva das contribuições relativas das atividades parassimpática e simpática para a VFC tornou-se possível por meio da análise no domínio da frequência, especificamente pela análise da densidade espectral de potência. Em condições experimentais controladas, a análise espectral da VFC é uma ferramenta valiosa para investigar os mecanismos neurais que regulam a FC.^24^

A análise no domínio da frequência da VFC — baseada no processamento matemático dos intervalos RR obtidos de registros de ECG em repouso — permite distinguir dois componentes espectrais principais: uma faixa de alta frequência (*high frequency* [HF], 0,15-0,40 Hz), considerada um marcador da atividade parassimpática, e uma faixa de baixa frequência (*low frequency* [LF], 0,04-0,15 Hz), tradicionalmente associada à modulação simpática.^24,25^ No entanto, algumas evidências sugerem que o componente LF reflete influências tanto simpáticas quanto vagais.^26,27^

De modo geral, a mensuração da VFC oferece uma abordagem não invasiva para avaliar a modulação do sistema nervoso autonômico em humanos, tanto em diferentes condições fisiológicas quanto em uma ampla variedade de estados fisiopatológicos clínicos.

Em nosso laboratório, séries temporais de intervalos RR foram obtidas por meio da digitalização de registros de ECG de Holter de 24 horas, com taxa de amostragem de 250 amostras por segundo, e armazenadas para análise computadorizada. Os dados foram coletados com o objetivo de avaliar os efeitos do treinamento aeróbico sobre a VFC durante a vigília e o sono, em homens saudáveis jovens e de meia-idade. A análise espectral de potência foi realizada utilizando o método autoregressivo ou a transformada rápida de Fourier (FFT). Para cada indivíduo, foram analisadas duas amostras com pelo menos 2 horas de duração: uma correspondente ao período de menor FC durante o sono noturno (refletindo maior atividade parassimpática) e outra ao período após o despertar (caracterizado por retirada parassimpática e aumento da atividade simpática). Foram documentadas duas faixas principais de frequência no espectro dos intervalos RR: LF (0,03-0,15 Hz) e HF (0,15-0,40 Hz). A razão entre os componentes LF e HF, comumente utilizada como índice de equilíbrio simpático-parassimpático, também foi calculada.^28,29^

### Cintilografia com 123I-MIBG para avaliação da inervação simpática miocárdica

As influências simpáticas no nível do miocárdio ventricular podem ser avaliadas de forma mais precisa por meio da cintilografia com ^123^I-MIBG, que permite examinar a inervação adrenérgica miocárdica. Em nosso laboratório, os estudos cintilográficos foram realizados utilizando uma câmera gama de cabeça dupla, equipada com detectores retangulares de campo de visão médio e colimadores de uso geral para baixa energia.

Os indivíduos foram avaliados em jejum, após um período de repouso de 1 hora, e após suspensão, por no mínimo 48 horas, de qualquer medicação que pudesse interferir na função adrenérgica. 1 mililitro de solução saturada de Lugol foi administrado por via oral uma hora antes da injeção do radiofármaco. Em seguida, um bolus intravenoso de 148 MBq de ^123^I-MIBG de alta atividade específica foi administrado, seguido de lavagem com 50 ml de solução salina, com o indivíduo em posição ortostática.

As imagens de tomografia computadorizada por emissão de fóton único foram adquiridas 2 horas após a injeção, utilizando uma órbita circular de 180° (do anterior direito ao oblíquo posterior esquerdo), com 32 projeções de 40 segundos cada. A aquisição foi realizada com matriz de 64 × 64 e tamanho de pixel de 0,6 cm. A discriminação energética foi obtida com uma janela simétrica de 20% centrada no pico fotoelétrico de 159 keV do ^123^I-MIBG. As imagens foram corrigidas quanto à uniformidade de campo e alinhamento do centro de rotação; os dados de projeção foram filtrados com filtro de Butterworth (potência = 5,0; frequência de corte = 0,25). Foram reconstruídos cortes tomográficos ortogonais padrão (eixo curto, eixo longo horizontal e eixo longo vertical). Para a análise das imagens, utilizou-se um modelo padronizado de 17 segmentos da parede do ventrículo esquerdo (VE), tanto em indivíduos saudáveis quanto em pacientes com doenças cardíacas.^30^

Os métodos padronizados descritos nesta revisão para avaliação do controle autonômico da FC em indivíduos saudáveis foram aplicados em nosso laboratório para investigar diversas condições clínicas (Tabela 1), incluindo cardiopatia chagásica, prolapso da válvula mitral, hipertireoidismo, pós-operatório cardíaco e insuficiência cardíaca. Também focamos em cenários fisiológicos, como treinamento de resistência e envelhecimento. Do ponto de vista clínico, o teste de inclinação tem se mostrado particularmente útil na avaliação de pacientes com diabetes, síncope e outras desordens neurológicas nas quais há suspeita de disfunção autonômica. Entre todos os métodos estudados, a imersão facial em água foi o único que não demonstrou reprodutibilidade suficiente para uso clínico rotineiro. Todas as demais abordagens apresentaram potencial de aplicação clínica em condições nas quais a regulação autonômica da FC desempenha papel crítico.

**Tabela 1:** – Efeitos esperados e interpretação de diversos testes de função autonômica

Teste	Efeitos esperados	Interpretação
Bloqueio farmacológico com atropina	Aumento da FC	Avalia a influência parassimpática sobre a FC
Bloqueio farmacológico com propranolol	Redução da FC	Avalia a influência simpática sobre a FC
Sensibilidade barorreflexa	Aumento ou redução transitória farmacologicamente induzida (20-30 mmHg) da PAS, com redução ou aumento reflexo da FC; permite correlação entre PAS e intervalo RR subsequente	A inclinação da linha de regressão indica a sensibilidade barorreflexa, refletindo o controle parassimpático da FC
Manobra de Valsalva	Após o fim do esforço expiratório, ocorre um aumento da PAS seguido de bradicardia reflexa abaixo do valor basal	A magnitude da bradicardia reflete a atividade do sistema nervoso parassimpático
Teste de inclinação a 70°	Resposta bifásica da FC: taquicardia inicial (nos primeiros 10 segundos) devido à retirada parassimpática; taquicardia sustentada (1-5 minutos) devido à ativação simpática	
Imersão facial em água	Bradicardia	A variabilidade das respostas provavelmente se deve à ativação simultânea de múltiplos receptores e vias aferentes. A resposta autonômica eferente resultante reflete a interação imprevisível desses mecanismos, o que limita a padronização do teste como método simples e reprodutível para avaliar a atividade parassimpática.
EI sustentado	Aumento rápido da FC no início da contração, com elevação progressiva durante o esforço sustentado	A regulação da FC segue um padrão bifásico: fase inicial mediada por retirada rápida do tônus parassimpático, seguida por ativação simpática significativa após 10 segundos de contração isométrica.
ED	Início rápido de taquicardia, seguido de aumento progressivo mais lento durante o esforço sustentado	A taquicardia induzida pelo exercício dinâmico é bifásica: começa com a retirada vaga (nos primeiros 10 segundos) e é seguida por ativação simpática progressiva, especialmente entre 1-4 minutos em cargas mais elevadas.
Arritmia sinusal respiratória	Variação da FC durante respiração padronizada a 6 ciclos por minuto com volume corrente de 1 litro	A VFC é mediada pela via eferente vagal
VFC	Análise espectral dos intervalos RR em repouso revela dois componentes principais: alta frequência (0,15-0,40 Hz) e baixa frequência (0,04-0,15 Hz)	O componente de alta frequência é um marcador da atividade parassimpática; o de baixa frequência sofre modulação simpática, embora algumas evidências sugiram influência combinada vagal e simpática.
Cintilografia com ^123^I-MIBG	Avaliação cintilográfica da inervação adrenérgica miocárdica	Demonstra a integridade da inervação simpática no nível ventricular do coração

^123^I-MIGB: ^123^I-metaiodobenzilguanidina; ED: exercício dinâmico; EI: exercício isométrico; FC: frequência cardíaca; PAS: pressão arterial sistólica; VFC: variabilidade da FC.

## References

[1] Hadaya J, Ardell JL (2020). Autonomic Modulation for Cardiovascular Disease. Front Physiol.

[2] Goldberger JJ, Arora R, Buckley U, Shivkumar K (2019). Autonomic Nervous System Dysfunction: JACC Focus Seminar. J Am Coll Cardiol.

[3] Marin-Neto JA, Pintya AO, Gallo L, Maciel BC (1991). Abnormal Baroreflex Control of Heart Rate in Decompensated Congestive Heart Failure and Reversal after Compensation. Am J Cardiol.

[4] Smyth HS, Sleight P, Pickering GW (1969). Reflex Regulation of Arterial Pressure during Sleep in Man. A Quantitative Method of Assessing Baroreflex Sensitivity. Circ Res.

[5] Pickering TG, Gribbin B, Petersen ES, Cunningham DJ, Sleight P (1972). Effects of Autonomic Blockade on the Baroreflex in Man at Rest and during Exercise. Circ Res.

[6] Levin AB (1966). A Simple Test of Cardiac Function Based Upon the Heart Rate Changes Induced by the Valsalva Maneuver. Am J Cardiol.

[7] Chow KE, Dhyani R, Chelimsky TC (2021). Basic Tests of Autonomic Function. J Clin Neurophysiol.

[8] Marin-Neto JA, Gallo L, Manco JC, Rassi A, Amorim DS (1980). Mechanisms of Tachycardia on Standing: Studies in Normal Individuals and in Chronic Chagas' Heart Patients. Cardiovasc Res.

[9] James JE, De Burgh Daly M (1972). Symp Soc Exp Biol.

[10] Finley JP, Bonet JF, Waxman MB (1979). Autonomic Pathways Responsible for Bradycardia on Facial Immersion. J Appl Physiol Respir Environ Exerc Physiol.

[11] Gallo L, Maciel BC, Manço JC, Marin-Neto JA (1988). Limitations of Facial Immersion as a Test of Parasympathetic Activity in Man. J Physiol.

[12] Lind AR, Taylor SH, Humphreys PW, Kennelly BM, Donald KW (1964). The Circulatory Effects of Sustained Voluntary Muscle Contraction. Clin Sci.

[13] Asmussen E (1981). Similarities and Dissimilarities between Static and Dynamic Exercise. Circ Res.

[14] Mitchell JH, Wildenthal K (1974). Static (Isometric) Exercise and the Heart: Physiological and Clinical Considerations. Annu Rev Med.

[15] Maciel BC, Gallo L, Marin-Neto JA, Martins LE (1987). Autonomic Nervous Control of the Heart Rate During Isometric Exercise in Normal Man. Pflugers Arch.

[16] Fagraeus L, Linnarsson D (1976). Autonomic Origin of Heart Rate Fluctuations at the Onset of Muscular Exercise. J Appl Physiol.

[17] Paulev PE (1971). Respiratory and Cardiac Responses to Exercise in Man. J Appl Physiol.

[18] Asmussen E, Johansen SH, Jørgensen M, Nielsen M (1965). On the Nervous Factors Controlling Respiration and Circulation during Exercise Experiments with Curarization. Acta Physiol Scand.

[19] Maciel BC, Gallo L, Marin-Neto JA, Lima EC, Martins LE (1986). Autonomic Nervous Control of the Heart Rate During Dynamic Exercise in Normal Man. Clin Sci.

[20] Gallo L, Maciel BC, Marin-Neto JA, Martins LE, Lima-Filho EC, Manço JC (1988). The Use of Isometric Exercise as a Means of Evaluating the Parasympathetic Contribution to the Tachycardia Induced by Dynamic Exercise in Normal Man. Pflugers Arch.

[21] Grossman P, Karemaker J, Wieling W (1991). Prediction of Tonic Parasympathetic Cardiac Control Using Respiratory Sinus Arrhythmia: The Need for Respiratory Control. Psychophysiology.

[22] Katona PG, Jih F (1975). Respiratory Sinus Arrhythmia: Noninvasive Measure of Parasympathetic Cardiac Control. J Appl Physiol.

[23] Maciel BC, Gallo J, Marin-Neto JA, Zanini-Maciel LM (1990). Depressed Respiratory Sinus Arrhythmia: Additional Evidence for Impairment of Vagal Activity in Human Hyperthyroidism. Braz J Med Biol Res.

[24] (1996). Heart Rate Variability: Standards Of Measurement, Physiological Interpretation and Clinical Use. Task Force of the European Society of Cardiology and the North American Society of Pacing and Electrophysiology. Circulation.

[25] Akselrod S, Gordon D, Ubel FA, Shannon DC, Berger AC, Cohen RJ (1981). Power Spectrum Analysis of Heart Rate Fluctuation: A Quantitative Probe of Beat-to-Beat Cardiovascular Control. Science.

[26] Skyschally A, Breuer HW, Heusch G (1996). The Analysis of Heart Rate Variability does not Provide a Reliable Measurement of Cardiac Sympathetic Activity. Clin Sci.

[27] Tiwari R, Kumar R, Malik S, Raj T, Kumar P (2021). Analysis of Heart Rate Variability and Implication of Different Factors on Heart Rate Variability. Curr Cardiol Rev.

[28] Emdin M, Marin-Neto JA, Carpeggiani C, Maciel BC, Macerata A, Pintya AO (1992). Heart Rate Variability and Cardiac Denervation in Chagas' Disease. J Ambulatory Monitor.

[29] Catai AM, Chacon-Mikahil MP, Martinelli FS, Forti VA, Silva E, Golfetti R (2002). Effects of Aerobic Exercise Training on Heart Rate Variability during Wakefulness and Sleep and cardiorespiratory Responses of Young and Middle-Aged Healthy Men. Braz J Med Biol Res.

[30] Simões MV, Pintya AO, Bromberg-Marin G, Sarabanda AV, Antloga CM, Pazin-Filho A (2000). Relation of Regional Sympathetic Denervation and Myocardial Perfusion Disturbance to wall Motion Impairment in Chagas' Cardiomyopathy. Am J Cardiol.

