# Supplementary material for: I - Avaliação da Função Autonômica Cardiovascular em Indivíduos Saudáveis
Source: Arq Bras Cardiol. 2026 Jan 9;122(12):e20250111. [Article in Portuguese] doi: 10.36660/abc.20250111 (PMC12978363; doi:10.36660/abc.20250111)
Supplement: Translation

# Translation: I - Evaluation of Cardiovascular Autonomic Function in Healthy Individuals

**DOI:** 10.36660/abc.20250111i

**Authors:** Benedito Carlos Maciel, Lourenço Gallo-Jr, André Schmidt, José Antonio Marin-Neto

**Affiliations:** 1 Brazil Laboratório de Hemodinâmica e Cardiologia Intervencionista, Hospital das Clínicas, Faculdade de Medicina de Ribeirão Preto, USP, São Paulo, SP – Brazil

**Keywords:** Autonomic Nervous System Diseases, Heart Rate, Sympathetic Nervous System, Parasympathetic Nervous System

## Abstract

This first part of the review presents nearly 60 years of experience from our laboratory in standardizing cardiovascular autonomic tests in healthy individuals. The wide range of tests includes pharmacological blockade of both the sympathetic and parasympathetic divisions, baroreflex sensitivity assessment through transient hypertension and hypotension, the Valsalva maneuver, head-up tilt testing, isometric and dynamic exercise, facial immersion in cold water, respiratory sinus arrhythmia, heart rate variability analysis in the frequency domain, and imaging with ^123^I-metaiodobenzylguanidine. Autonomic function was evaluated based on test responses under baseline conditions in healthy individuals. The analysis also covers physiological adaptations to endurance training and aging, providing a reference framework for identifying autonomic dysfunction involving both parasympathetic and adrenergic components in patients with various pathophysiological conditions, as discussed in the second part of this review.

## Introduction

In healthy individuals, a complex anatomical and functional autonomic neural network regulates cardiac chronotropic, lusitropic, dromotropic, and inotropic functions.^1,2^ This first part of the review compiles findings from studies conducted in healthy individuals. The knowledge derived from these studies forms the foundation for understanding how dysfunction of the cardiovascular autonomic nervous system contributes to the pathophysiology of numerous clinical conditions. The findings related to such dysfunctions under abnormal conditions are discussed in the second part of this publication.

In this review, we draw on over 55 years of expertise from our laboratory using standardized tests to assess cardiac autonomic physiology in healthy individuals and autonomic dysfunction in humans with various pathophysiological conditions. While cardiovascular autonomic function can be evaluated through direct nerve recordings or imaging-based techniques — such as ventricular uptake of ^131^I-meta-iodobenzylguanidine — our work has primarily focused on heart rate (HR) responses to several stimuli mediated by autonomic influences on the sinus node.

The broad range of tests examined different aspects of cardiac autonomic control under resting conditions, during pharmacological blockade of the parasympathetic and/or sympathetic branches of the autonomic nervous system, and in response to standardized physiological maneuvers, as described below.

Across the different studies, the groups of healthy individuals ranged from 8 to 23 individuals, with an average age between 27 and 31 years (age range: 19 to 43 years). All participants were male and considered healthy after a comprehensive clinical evaluation. None were taking any medications at the time of testing.

### Functional tests for evaluating cardiovascular autonomic function

To assess cardiovascular autonomic function in healthy individuals, we standardized the use of tests under baseline resting conditions and following pharmacological blockade of the efferent parasympathetic and sympathetic branches of the autonomic nervous system (Central Illustration). These tests also included evaluating HR reflex responses to both physiological and pharmacological changes in systolic arterial pressure (SAP).

Briefly, after baseline HR and SAP were recorded with the individual in a supine position (typically after 20 minutes of rest), the same parameters were measured following intravenous administration of either: a) atropine sulfate (0.04 mg/kg, infused slowly at 0.25 mg/min); or b) propranolol hydrochloride (0.2 mg/kg, infused slowly at 1 mg/min).

In most studies, HR and SAP were also recorded after the combined administration of intravenous atropine and propranolol, allowing for the determination of intrinsic sinus node HR. The physiological maneuvers described in the following sections of this review were conducted before and after each of these three types of pharmacological autonomic blockade.

### Evaluating baroreflex control of HR: phenylephrine, amyl nitrite, and Valsalva maneuver^3^

All tests were conducted with participants in the supine position. Under local anesthesia, an indwelling catheter was inserted into an antecubital vein, and a 20G Cournand needle was placed into the brachial artery. An intravenous bolus of 25 to 400 µg of phenylephrine hydrochloride was administered, followed by a 10 mL saline flush, to induce an increase of 20 to 30 mmHg in SAP. For the amyl nitrite test, the contents of two ampoules (0.2 mL each, 96% amyl nitrite) were inhaled over two respiratory cycles, aiming to reduce SAP by 10 to 30 mmHg below baseline.

Baroreflex sensitivity was assessed by analyzing the acute reflex changes in HR triggered by transient variations in SAP, as described below.

The Valsalva maneuver was performed through a forced expiration against resistance, ensuring that the glottis remained open during the strain. The expiratory pressure was maintained at 40 mmHg for 20 seconds. During the phenylephrine, amyl nitrite, and Valsalva tests, electrocardiography (ECG), SAP, and oral pressure were continuously recorded at a paper speed of 25 mm/s.

Data obtained from the phenylephrine test were analyzed by selecting, for each individual, only those injections that produced regression lines correlating each SAP value with the subsequent RR interval on the ECG, provided the correlation coefficients were statistically significant (p < 0.05). The slopes of 3 to 4 regression lines were averaged to yield the mean baroreflex sensitivity for each individual, following the method described by Smith, Sleight et Pickering.^4^ Baroreflex sensitivity during the amyl nitrite test was quantified by plotting the sequentially decreasing SAP values against the corresponding RR intervals of the next cardiac cycle. The average of three tests was calculated for each individual.^4-6^

Changes in SAP during the Valsalva maneuver were continuously recorded throughout all phases of the test. The maneuver produces a sudden reduction in cardiac preload due to decreased venous return, resulting in a fall in SAP and triggering compensatory mechanisms such as arterial vasoconstriction and tachycardia during the expiratory strain. In healthy individuals, partial recovery of SAP typically occurs during the strain phase.^7^ The chronotropic response was expressed as the Valsalva ratio, calculated by dividing the longest RR interval during phase IV (post-strain, when pressure overshoot occurs) by the shortest RR interval observed during phase II (the hypotensive phase of the expiratory effort). The mean of three tests was recorded for each individual.^6^

All tests were performed before and after intravenous administration of atropine sulfate, as previously described. The extent of the HR increase following atropine administration reflects the level of tonic vagal restraint on the sinus node.

Additional insight into parasympathetic cardiac influence is provided by baroreflex sensitivity values measured during transient hypertension and hypotension induced by pharmacological agents — phenylephrine and amyl nitrite, respectively. During the Valsalva maneuver, parasympathetic autonomic function is primarily assessed by the bradycardia that occurs immediately after release of the expiratory strain (phase IV), which leads to a transient HR reduction below baseline. Notably, the early HR increase observed during phase II is not driven by sympathetic activation, but rather by parasympathetic withdrawal.^7^ The vasomotor adrenergic response is reflected in the recovery of blood pressure during phases III and IV of the Valsalva maneuver. In parallel with parasympathetic withdrawal, the HR increase during the strain phase (phase II) also represents the intensity of sympathetic activation acting on the sinus node.^3^

### Tilting test to evaluate HR control by the parasympathetic and adrenergic systems

To assess the hemodynamic effects of acute orthostatic stress on HR and SAP regulation, healthy individuals were rapidly and passively tilted to a 70° head-up position after resting in the supine position for 20 minutes. A specialized tilt table was used to eliminate any muscular effort regardless of body position. Instantaneous HR was monitored using a linear rate meter triggered by a standard ECG lead, with both signals displayed and recorded in real time via an online acquisition system. SAP was continuously recorded following percutaneous insertion of a Cournand needle into either the brachial or femoral artery, connected to a pressure strain-gauge transducer.^8^ Hemodynamic parameters were recorded while individuals remained passively upright at 70° for 5 minutes.

The procedure was then repeated following intravenous administration of atropine sulfate (0.04 mg/kg, infused slowly at 0.25 mg/min). In a subsequent session, conducted on a different day after the effects of atropine had subsided, the postural shift to the upright position was repeated under the effect of propranolol hydrochloride (0.2 mg/kg, infused slowly at 1 mg/min). Within 60 minutes of propranolol administration, atropine was administered again as previously described. A third tilting test was then performed to assess the hemodynamic responses to postural stress under double autonomic blockade — i.e., with intrinsic sinus node HR in effect, free of autonomic modulation.

Orthostatic stress causes acute pooling of blood in the lower body venous system, whether individuals actively assume the upright posture or are passively tilted to a 70° head-up position. This maneuver leads to decreased venous return to the heart and a rapid 15-25% reduction in stroke volume and cardiac output. In healthy individuals, physiological compensatory mechanisms include reflex tachycardia and systemic arteriolar vasoconstriction. Following a brief, mild decrease in SAP, a hemodynamic steady state is typically established, with mean SAP maintained at levels similar to those observed in the supine position. The reduction in stroke volume is thus compensated by an increase in HR and enhanced systemic vascular resistance.^7,8^

In healthy individuals, a significant increase in HR — approximately 15 to 25 beats per minute — is observed within the first 10 seconds after passive tilting to the upright position. This initial response is mediated by parasympathetic withdrawal, as it is abolished following atropine administration. Beta-adrenergic blockade does not significantly affect this early (10-second) HR increase; however, it markedly reduces the HR elevation observed at 1 and 5 minutes of upright tilt in healthy individuals. These findings suggest a biphasic tachycardic response to orthostatic stress: the initial phase is driven primarily by parasympathetic withdrawal, while sympathetic activation becomes the predominant mechanism once hemodynamic stabilization is achieved in the upright position.

### Facial water immersion as a test of parasympathetic HR control

Previous studies have demonstrated that the bradycardia induced by diving or facial immersion is mediated by the efferent parasympathetic pathway, as it can be abolished by either surgical vagotomy or atropine administration.^9,10^ While several receptors and afferent pathways may contribute to this response, the relative impact of individual stimuli — such as immersion itself, water temperature, pulmonary volume, apnea, and intrathoracic pressure — has not been fully elucidated.

In our laboratory, we investigated the HR response to facial immersion in water, both as an isolated maneuver and in combination with apnea, in healthy participants. The goal was to define the conditions under which this response could serve as a standardized, simple, noninvasive, and reproducible test of parasympathetic activity.

The following procedures were evaluated:^11^ (a) (a) 10-second apnea in air at various lung volumes; (b) facial immersion in water for 2 minutes at different temperatures (5°C, 15°C, and 25°C), with respiration maintained via a closed air circuit; and (c) a combination of facial immersion and apnea at different lung volumes. A subset of healthy participants was re-evaluated following pharmacological parasympathetic blockade with atropine sulfate (0.04 mg/kg body weight).

Our main findings were as follows: (a) apnea in air elicited HR responses that were dependent on lung volume; (b) facial immersion in water induced transient bradycardia, with a peak effect observed between 20 and 30 seconds of immersion; (c) water temperature had no appreciable effect on the magnitude of bradycardia; (d) the combination of immersion and apnea produced heterogeneous HR responses, without any potentiation of bradycardia compared to each maneuver performed separately; and (e) atropine failed to reduce the magnitude of bradycardia induced by facial immersion in two of the three individuals re-evaluated after parasympathetic blockade.

Variability in responses in this study was likely due to the simultaneous stimulation of multiple receptors and afferent pathways during the maneuvers. As a result, the autonomic efferent response depends on the complex and unpredictable net interaction among these mechanisms. This variability represents a limiting factor in the standardization of facial immersion as a simple and reproducible method for assessing parasympathetic activity.^11^

Moreover, the results obtained under pharmacological blockade suggest that the vagal efferent pathway is not the sole contributor to the bradycardia induced by facial immersion without apnea.^11^

### Autonomic regulation of HR during isometric exercise (IE) in healthy individuals

The HR response pattern to sustained IE has been extensively studied and is well characterized in humans, based on previous research corroborated by our own findings. HR increases very rapidly at the onset of isometric contraction, typically within the first 500 milliseconds of exertion. The magnitude of this increase appears to be directly proportional to the relative level of muscle tension generated — i.e., the percentage of maximum voluntary contraction (MCV) that the individual can sustain for a limited period using a specific muscle group (e.g., upper or lower limb). When the contraction is maintained, HR continues to rise progressively, particularly at higher levels of muscle tension.^12^

The relative contribution of each efferent branch of the autonomic nervous system to the tachycardia induced by IE was evaluated in healthy individuals using the handgrip method. The exercise was performed at the MVC — defined as the greatest level of tension an individual could sustain for 10 seconds — and at submaximal levels of 75%, 50%, and 25% of MVC, sustained for 20, 40, and 10 seconds, respectively.^15^ Individuals were seated, and the right hand was positioned to grip the handle of an isometric dynamometer. Respiratory patterns were closely monitored to ensure spontaneous, regular breathing through a mouthpiece connected to a Hans Rudolph-type valve, with nasal occlusion maintained using a nose clip. The MVC (considered 100%) was established for each individual and used to calibrate the other intensity levels tested.

The study was conducted both under baseline conditions and after pharmacological blockade with atropine or propranolol.^15^ Under control conditions, HR responses to IE were intensity- and duration-dependent, showing a progressive increase during sustained contraction. The tachycardia was of rapid onset and substantial magnitude, particularly at higher intensities of handgrip.^15^ Parasympathetic blockade significantly attenuated the HR increase observed during the first 10 seconds of exercise across all intensity levels (Figure 1). In contrast, sympathetic blockade with propranolol markedly blunted the HR response after 10 seconds of effort at 75% and 50% MVC (Figure 2). A mild reduction in tachycardia could also be observed after just 10 seconds of maximal exertion under sympathetic blockade. These findings indicate that autonomic regulation of HR during IE follows a biphasic pattern: the initial response is primarily mediated by rapid parasympathetic withdrawal, followed by a prominent sympathetic contribution that becomes more apparent after 10 seconds of sustained contraction — or even slightly earlier at maximal intensity.^15^

**Figure 1:** – Heart rate responses to isometric exercise at 100%, 75%, 50%, and 25% of maximum voluntary contraction in healthy individuals, during 10, 20, 40, and 10 seconds of effort, respectively, recorded under control conditions and after pharmacological parasympathetic blockade with atropine (0.04 mg/kg body weight; n = 12). Values are expressed as mean ± SEM at 10-second intervals. The exercise period is indicated by vertical bars.15

**Figure 2:** – Heart rate responses to isometric exercise at 100%, 75%, 50%, and 25% of maximum voluntary contraction in healthy individuals, during 10, 20, 40, and 10 seconds of effort, respectively, recorded under control conditions and after pharmacological sympathetic blockade with propranolol (0.2 mg/kg body weight; n = 11). Values are presented as mean ± SEM at 10-second intervals. The exercise period is indicated by vertical bars.15

### Autonomic regulation of HR during dynamic exercise (DE) in healthy individuals

Previous studies have shown that the onset of DE is accompanied by a rapid increase in HR. The abrupt nature of this tachycardia suggests a neurogenic regulatory mechanism, with the efferent response predominantly mediated by vagal withdrawal at the level of the sinus node. Following this initial phase, HR continues to rise more gradually.^16-18^ In our investigations involving healthy male individuals, we assessed the relative contributions of the sympathetic and parasympathetic branches of the autonomic nervous system to the HR response induced by DE. The exercise test was performed both before and after pharmacological blockade with either atropine or propranolol. DE was performed in the seated position by using a calibrated electromagnetic bicycle ergometer. Individuals exercised at workloads of 25, 50, and 100 watts — each sustained for 4 minutes. Variable rest intervals were provided between effort levels to allow HR to return to baseline. Pedaling speed was maintained at a constant rate of 60 revolutions per minute. ECG and instantaneous HR were continuously recorded from 30 seconds before exercise initiation to 1 minute after its completion.

Parasympathetic blockade significantly attenuated the rapid HR response at the onset of exercise across all intensity levels, whereas sympathetic blockade primarily affected the slower HR increase during the 1-4 minute phase, particularly at the highest workload^19^ (Figure 3). These findings support the hypothesis that the tachycardia induced by DE is mediated by a biphasic mechanism: an initial phase driven by rapid vagal withdrawal, followed by a gradual increase in sympathetic activity, which becomes more pronounced at higher exercise intensities.

**Figure 3:** – Heart rate increments (ΔHR) induced by dynamic exercise in healthy individuals at workloads of 25, 50, and 100 watts, measured at the following time intervals: 0-10 s, 0-30 s, 30-60 s, and 1-4 min. Bars represent mean ± SEM values recorded before (open bars) and after pharmacological blockade with atropine (0.04 mg/kg body weight; n = 13, closed bars) or propranolol (0.2 mg/kg body weight; n = 10, hatched bars). NS: not significant.19

Therefore, continuous HR monitoring throughout the entire period of DE, across different intensity levels, provides a simple, noninvasive method for the functional evaluation of both parasympathetic and sympathetic components of cardiac autonomic regulation.^19^

### Studies combining DE and IE in healthy individuals

Our investigations also included healthy individuals performing combined IE and DE. This study exemplifies our laboratory’s approach to integrating multiple autonomic tests to better understand human physiological responses. The primary aim was to use IE as a tool to evaluate the mechanisms underlying HR increases during DE.

The intensity of the IE (handgrip) was standardized to elicit an HR increase primarily through vagal withdrawal. IE was performed using a strain-gauge dynamometer with a linear response at 75% of MVC, sustained for 10 seconds and repeated at 1-minute intervals. HR responses to IE were assessed under baseline conditions, during DE, and throughout the corresponding recovery phase.

DE was performed using the lower limbs on a seated electromagnetic bicycle ergometer for 4 minutes at workloads of 55 and 105 watts, with rest intervals between efforts. In the combined protocol, IE was applied at the onset of DE, at 1, 2, and 3 minutes during DE, and at 0, 1, 2, 3, and 5 minutes into the recovery phase.

The following results were observed: (1) IE performed during DE consistently produced a smaller increase in HR compared to IE alone. This effect was more pronounced at 105 W than at 55 W, suggesting that vagal withdrawal occurred at the very onset of DE and was sustained throughout the effort in a workload-dependent manner; (2) during DE, IE continued to elicit additional HR increases — even at the 105 W workload, where HR was already rising gradually due to DE alone — indicating that the progressive tachycardia observed between the first and fourth minute of DE was not attributable to vagal withdrawal; (3) The standardized IE protocol enabled the functional assessment of vagal withdrawal dynamics during DE without the need for pharmacological autonomic blockade.^20^

### Respiratory sinus arrythmia as a test of autonomic HR control

Since HR fluctuations during respiratory sinus arrhythmia are primarily mediated by the vagal efferent pathway,^21,22^ we standardized this method as a simple and quantifiable technique for evaluating parasympathetic activity.^23^ In this test, individuals were seated and instructed to breathe into a closed-circuit spirometer equipped with a linear potentiometer. This setup allowed simultaneous recording of respiratory volume using a thermal recorder and one channel of a storage oscilloscope.

The second oscilloscope channel was connected to a function generator that produced a sinusoidal wave of predetermined amplitude and frequency. Individuals were instructed to match their breathing pattern to the sinusoidal signal generated. The test was conducted at a frequency of 6 cycles per minute, with the tidal volume adjusted to 1 liter. This standardization reliably elicited marked respiratory sinus arrhythmia in healthy individuals.

Throughout the procedure, ECG, instantaneous HR, and respiratory tidal volume were continuously recorded. For each individual, mean HR variability (HRV) was calculated as the difference between the maximum and minimum instantaneous HR across nine consecutive respiratory cycles.

### HRV as a test of autonomic function

In healthy individuals, HRV primarily reflects the efferent autonomic modulation of the sinus node. For many years, HRV was expressed solely through time-domain measures, such as mean values and standard deviations, which quantify variability based on RR interval fluctuations. However, the noninvasive assessment of the relative contributions of parasympathetic and sympathetic activity to HRV became possible through frequency-domain analysis, specifically via power spectral density analysis. Under controlled experimental conditions, spectral analysis of HRV is a valuable tool for investigating the neural mechanisms regulating HR.^24^

Frequency-domain analysis of HRV — based on mathematical processing of RR intervals from ECG recordings obtained at rest — can distinguish two primary spectral components: a high-frequency (HF) band (0.15-0.40 Hz), considered a marker of parasympathetic activity, and a low-frequency (LF) band (0.04-0.15 Hz), traditionally associated with sympathetic modulation.^24,25^ However, some evidence suggests that the LF component reflects both sympathetic and vagal influences.^26,27^

Overall, HRV measurement provides a noninvasive approach to evaluate autonomic nervous system modulation in humans under various physiological conditions, as well as in a wide range of clinical pathophysiological states.

In our laboratory, time series of RR intervals were obtained by digitizing 24-hour Holter ECG recordings at a sampling rate of 250 samples per second and storing them for computerized analysis. Data were collected to evaluate the effects of aerobic exercise training on HRV during wakefulness and sleep in young and middle-aged healthy men. Power spectral analysis was performed using either the autoregressive method or the fast Fourier transform. For each individual, two samples of at least 2 hours in duration were analyzed: one corresponding to the period with the lowest HR during nighttime (reflecting enhanced parasympathetic activity), and the other corresponding to the post-awakening period (characterized by parasympathetic withdrawal and increased sympathetic activity). Two major frequency bands in the RR interval spectrum were documented: LF (0.03-0.15 Hz) and HF (0.15-0.40 Hz). The ratio of LF to HF power, commonly used as an index of sympathetic-to-parasympathetic balance, was also calculated.^28,29^

### 123I-metaiodobenzylguanidine (123I-MIBG) scintigraphy for evaluation of myocardial sympathetic innervation

Sympathetic influences at the level of the ventricular myocardium can be more precisely assessed using ^123^I-MIBG scintigraphy, which evaluates myocardial adrenergic innervation. In our laboratory, scintigraphy studies were performed using a dual-head gamma camera equipped with rectangular medium field-of-view detectors and low-energy, all-purpose collimators.

Individuals were studied under fasting conditions, following a 1-hour rest period, and after discontinuation of any medications that could interfere with adrenergic function for at least 48 hours. 1 milliliter of saturated Lugol’s solution was administered orally 1 hour prior to radiotracer injection. A bolus of 148 MBq of high-specific-activity ^123^I-MIBG was then injected intravenously, followed by a 50 mL saline flush, with individuals in the upright position.

Single-photon emission computed tomography images were acquired 2 hours post-injection, using a 180° circular orbit (from right anterior to left posterior oblique), with 32 projections at 40 seconds per projection. Acquisition was performed using a 64 × 64 matrix with a pixel size of 0.6 cm. Energy discrimination was achieved with a 20% symmetric window centered at the 159 keV photopeak of ^123^I-MIBG. Images were corrected for field uniformity and center-of-rotation alignment; projection data were filtered using a Butterworth filter (power = 5.0, cutoff frequency = 0.25). Standard orthogonal tomographic slices (short-axis, horizontal long-axis, and vertical long-axis) were reconstructed. A standardized 17-segment left ventricular (LV) wall model was used for image analysis in both healthy individuals and patients with disease.^30^

The standardized methods described in this review for evaluating autonomic control of HR in healthy individuals were applied in our laboratory to investigate various clinical conditions (Table 1), including Chagas heart disease, mitral valve prolapse, hyperthyroidism, post-cardiac surgery, and heart failure. We also focused on physiological scenarios such as endurance training and aging. From a clinical perspective, the tilting test has been particularly useful in the evaluation of patients with diabetes, syncope, and other neurological disorders in which autonomic dysfunction is suspected. Among all the methods studied, facial immersion in water was the only one that did not demonstrate sufficient reproducibility for routine clinical use. All other approaches showed potential for clinical application in conditions where autonomic regulation of HR plays a critical role.

**Table 1:** – Expected effects and interpretation of several tests of autonomic function

Test	Expected Effects	Interpretation
Pharmacological blockade with atropine	Increase in HR	Assesses parasympathetic influence on HR
Pharmacological blockade with propranolol	Decrease in HR	Assesses sympathetic influence on HR
Baroreflex sensitivity	Pharmacologically induced transient increase or decrease (20-30 mmHg) in SAP, with reflex decrease or increase in HR; allows correlation of each SAP value with the subsequent RR interval	Slope of the regression line indicates baroreflex sensitivity, reflecting parasympathetic control of HR
Valsalva maneuver	After release of expiratory strain, SAP overshoot is followed by reflex bradycardia below baseline	Magnitude of bradycardia reflects parasympathetic nervous system activity
70° head-up tilt test	Biphasic HR response: initial tachycardia (first 10 seconds) due to parasympathetic withdrawal; sustained tachycardia (1-5 minutes) due to sympathetic activation	
Facial water immersion	Bradycardia	Response variability is likely due to simultaneous activation of multiple receptors and afferent pathways. The resulting autonomic efferent output reflects the unpredictable net interaction of these mechanisms, limiting the test’s standardization as a simple and reproducible method for assessing parasympathetic activity.
Sustained IE	Rapid increase in HR at the onset of contraction, with progressive elevation during sustained effort	HR regulation follows a biphasic pattern: the initial phase is driven by rapid parasympathetic withdrawal, followed by significant sympathetic activation after 10 seconds of isometric contraction.
DE	Rapid onset of tachycardia followed by a slower progressive increase during sustained effort	Tachycardia induced by dynamic exercise is biphasic: it begins with vagal withdrawal (within 10 seconds) and is followed by progressive sympathetic activation, especially during the 1-4 minute period at higher workloads.
Respiratory sinus arrhythmia	HR variation during standardized breathing at a frequency of 6 cycles per minute with a tidal volume of 1 liter	HRV is mediated by the efferent vagal pathway.
HRV	Frequency-domain analysis of RR intervals under resting conditions reveals two main spectral components: high frequency (0.15-0.40 Hz) and low frequency (0.04-0.15 Hz)	The high-frequency component is a marker of parasympathetic activity; the low-frequency component is influenced by sympathetic modulation, though some evidence suggests combined vagal and sympathetic contributions.
^123^I-MIBG scintigraphy	Scintigraphy assessment of myocardial adrenergic innervation	Demonstrates the integrity of sympathetic innervation at the ventricular level of the heart

^123^I-MIGB: ^123^I-metaiodobenzylguanidine; HR: heart rate; SAP: systolic arterial pressure; IE: isometric exercise; DE: dynamic exercise; HRV: heart rate variability.
